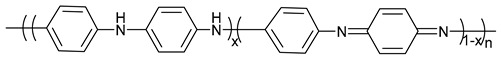# Flexible Organic Thermoelectric Materials and Devices for Wearable Green Energy Harvesting

**DOI:** 10.3390/polym11050909

**Published:** 2019-05-20

**Authors:** Yinhang Zhang, Soo-Jin Park

**Affiliations:** Department of Chemistry, Inha University, 100 Inharo, Incheon 22212, Korea; bank0719@163.com

**Keywords:** organic thermoelectric materials, Seebeck coefficient, thermal conductivity

## Abstract

In the past few decades, organic thermoelectric materials/devices, which can exhibit remarkable potential in green energy conversion, have drawn great attention and interest due to their easy processing, light weight, intrinsically low thermal conductivity, and mechanical flexibility. Compared to traditional batteries, thermoelectric materials have high prospects as alternative power generators for harvesting green energy. Although crystalline inorganic semiconductors have dominated the fields of thermoelectric materials up to now, their practical applications are limited by their intrinsic fragility and high toxicity. The integration of organic polymers with inorganic nanoparticles has been widely employed to tailor the thermoelectric performance of polymers, which not only can combine the advantages of both components but also display interesting transport phenomena between organic polymers and inorganic nanoparticles. In this review, parameters affecting the thermoelectric properties of materials were briefly introduced. Some recently developed n-type and p-type thermoelectric films and related devices were illustrated along with their thermoelectric performance, methods of preparation, and future applications. This review will help beginners to quickly understand and master basic knowledge of thermoelectric materials, thus inspiring them to design and develop more efficient thermoelectric devices.

## 1. Introduction

Flexible wearable electronic devices have attracted great interest and have gradually emerged in daily life due to their light weight, easy skin attachment potential and the ability to withstand mechanical deformation [1,2,3,4,5,6,7,8]. The devices currently in use generally require externally powered drives, which greatly limit the advantages in wearability. The emergence of organic thermoelectric materials essentially addresses this issue owing to their unique ability in converting heat difference to electric voltage [9,10,11,12,13,14,15,16,17].

Thermoelectric materials are designed according to the thermoelectric effect, known as the Seebeck effect, which was originally discovered by Alessandro Volta in 1794 and rediscovered and named by Thomas Johann Seebeck in 1821 [18,19,20]. Thermoelectric devices are playing an increasingly crucial role in harvesting green energy for future wearable electronic devices owing to the fact that they can produce energy without shifting mechanical components, hence, guaranteeing high reliability [21,22,23,24,25,26]. The thermoelectric material efficiency is evaluated by a dimensionless figure-of merit, *ZT* = *S*^2^*σTκ*^−1^, where the *κ* is the thermal conductivity, *σ* is the electrical conductivity, and *S* is the Seebeck coefficient [27,28,29,30]. The strong interdependence of these three parameters, introduced in the following section, makes optimization to obtain a high ZT value challenging. In the case of organic thermoelectric materials, owing to their intrinsically low thermal conductivity, their thermoelectric efficiency can be expressed in a new criterion called the power factor, *PF* = *S*^2^*σ* [31,32,33,34]. 

Thermoelectric materials contain traditional semiconductor-based inorganic and recently developed polymer-based organic materials. Inorganic thermoelectric materials, such as SnSe [35,36,37], PbTe [38,39,40], CuS [41], PbS [42,43,44], PbSe [45,46], Bi_2_Te_3_ [47,48], Te [49], BiSbTe [50], and SnTe [51] exhibited higher thermoelectric efficiency than those of the organic thermoelectric materials. Zhao et al. [35] developed SnSe single crystals with an unprecedented ZT value of 2.6 ± 0.3 at 923 K measured along the *b*-axis of the room temperature orthorhombic unit cell. Along the *c*-axis, the ZT value was measured to be 2.3 ± 0.3. The super-high ZT value was attributed to the ultralow lattice thermal conductivity of SnSe caused by anharmonicity. Wu et al. [52] reported that 2.5% K-doped PbTe_0.7_S_0.3_ achieves a ZT value of >2 with the temperature ranging from 673 to 923 K and has a relatively high average ZT value of 1.56 in the temperature region of 300–900 K. The author attributed the high ZT value to the synergistically optimized thermal and electrical transport properties, hierarchical architecting, and band-structure engineering. Although most of the inorganic thermoelectric materials yield ZT values greater than 1, their applications are limited on account of their intrinsic demerits, such as their heavy weight, high cost, processing difficulty, and toxicity. 

Conversely, polymer-based organic thermoelectric generators, which contain unique merits such as low cost, light weight, convenient processing, mechanical flexibility, and low thermal conductivity, have been promoted as a new generation of thermoelectric candidates [53,54,55,56,57,58,59]. However, these organic thermoelectric devices are still not practically employed or applied owing to their low thermoelectric efficiency or ZT value. With effort, great progress has been achieved, and some recently developed organic thermoelectric materials have exhibited similar thermoelectric performance to those of traditional inorganic thermoelectric materials. Non-conducting polymers and conducting polymers are both used to prepare organic thermoelectric devices, but conducting polymers play the dominant role. Poly(3-hexylthiophene) (P3HT) [60], poly(3-octylthiophene) [61], and polyvinylidene fluoride [62,63] are three commonly used non-conducting polymers, while conducting polymers include poly(3-methylthiophene) [64,65], polyacetylene [66,67], poly(aniline) [68,69], polypyrrole [34,70], polythiophenes [58,71], polyphenylenevinylene [72], and poly(3,4-ethylenedioxythiophene) [73,74,75,76], and their chemical structures are shown in Table 1.

In this review, we introduced the effect of parameters on the thermoelectric efficiency and summarized the recently studied organic thermoelectric materials. In our previously published review paper, carbon-based thermoelectric materials were mainly introduced [31]. Carbon materials are usually non-toxic, light, and environmentally friendly, with excellent reinforcement ability [77,78,79,80,81,82,83,84,85,86,87,88,89,90]. This review gives more attention to summarizing inorganic particle-based organic thermoelectric materials that have been developed in recent years. Their designing philosophy and unique performance were sketched, and the future outlook of thermoelectric materials was briefly presented. 

## 2. Parameters Affecting Thermoelectric Efficiency 

As introduced in the last section, the ability of a thermoelectric device to generate thermoelectric power can be evaluated by the dimensionless figure of merit, *ZT* = *S*^2^*σTκ*^−1^. Consequently, the Seebeck coefficient *S*, thermal conductivity *κ*, and electrical conductivity *σ* are three inherent and also predominant parameters that essentially affect the thermoelectric efficiency of a given material. To obtain ideal thermoelectric energy conversion efficiency, thermoelectric materials with a high Seebeck coefficient, high electrical conductivity, and low thermal conductivity are required at the specified temperature [91,92,93,94,95,96]. However, it is extremely difficult to optimize the thermoelectric efficiency by independently adjusting these three parameters, as these three parameters display a triangular relationship. Thus, the adjustment should be judiciously optimized to obtain high-performance thermoelectric devices. Up to now, the highest ZT value of the available materials achieved is approximately 1 at room temperature. 

As introduced, the Seebeck coefficient (*V*/*K*) of a thermoelectric material is the magnitude of an induced thermoelectric voltage in response to different temperatures at both sides of the material. It can be defined by the equation, *S* = −*ΔV*/*ΔT*, where *ΔV* is the thermoelectric voltage, and *ΔT* is the temperature difference between the two ends of the thermoelectric material. Thermoelectric materials charged with negative carriers, such as electrons, exhibit a negative Seebeck coefficient, while materials charged with positive carriers, such as holes, exhibit a positive Seebeck coefficient [97,98,99,100]. The Seebeck coefficient of a specified material is not stable at different external temperatures, but at a specific temperature, it depends on the chemical composition of the materials. In quantum theory, the Seebeck coefficient is an entropy measure for a carrier with a unit charge [101,102]. 

Electrical conductivity, which is positively correlated with thermoelectric efficiency, is another crucial factor in obtaining higher thermoelectric efficiency. It can be expressed by the equation σ = μqn, where q is the elementary charge, n is the charge carrier density, and μ is the electron mobility [103,104]. This equation applies to both the electron-flow-based n-type semiconductors and hole-flow-based p-type semiconductors. The charge carrier density is the number of charge carriers per volume. The hole or electron mobility is determined by the scattering time. Ionized impurity scattering caused by ionization occurring in the lattice and acoustic phonon scattering occurring during phonon collisions in a non-linear interaction are the most crucial scattering sources [31,105].

Thermal conduction is achieved by the transfer of a particle’s vibration energy to its adjacent particles without matter motion, which takes place mainly through collision [106] The thermal conductivity (k) is realized by the phonon synergy (k_L_) and electron transport (k_e_), k = k_L_ + k_e_. In solid materials, such as in conductors and polymer composites, thermal conduction is the main method of heat transfer. However, the mechanism for thermal conduction in solids is complex, requiring the consideration of many parameters, such as size, proportion, thickness, morphological aspects, defects, orientation and alignment, and interfacial thermal resistance, and their effect on thermal conduction has been comprehensively illustrated in our previous studies [107,108,109]. In perfectly crystalline and rigid samples, thermal conduction can be elucidated by the following visualized phenomenon: the atoms on one side of the material obtain thermal energy, and the vibrational energy is transferred to the adjacent atoms via diffusion with a common vibrational mode through the entire crystal. In amorphous and disordered polymers, the thermal conduction mechanism is much more complex. The heat energy is first absorbed by the surface atoms and then transferred to the adjacent ones in a much slower speed, which usually causes disordered rotations and vibrations of other atoms around their equilibrium position, followed by scattering to its adjacent chains [106]. In crystalline particle-reinforced polymer composites, the thermal conduction mechanism is even more complex and does not involve just the simple addition of conduction in a crystalline particle and in a disordered polymer. Additional factors such as the concentration of the crystalline particle, particle dispersion, particle networks in the polymer matrix, processing, particle alignment, and interfacial thermal resistance should be considered. 

## 3. Recently Developed Thermoelectric Materials

### 3.1. Recently Developed p-Type Organic Thermoelectric Materials

Holes and electrons are two typical carriers in thermoelectric materials, and holes are the dominant carriers in p-type organic thermoelectric materials. Under specific conditions, CNT [110,111], bismuth antimony telluride [112], Bi_2_S_3_ [113], Bi_2_Te_3_ [114], and graphene (oxide) [33,115,116] are some of the most used p-type thermoelectrical materials. In this section, some recently developed p-type thermoelectric materials were briefly introduced.

In the study of Cho et al. [21], ordered polyelectrolyte nanocomposites were deposited through layers of polyaniline (PANi), graphene, and double-walled CNT (DWNT) using a layer-by-layer assembly. The resistance of PANi/DWNT and PANi/graphene decreased as a function of deposited bilayers because more layers can provide more efficient electron transport. With 40 bilayers, the electrical conductivity of PANi/DWNT reached 840 S/cm. In contrast, PANi/graphene with 40 bilayers exhibited a relatively low electrical conductivity of 0.14 S/cm because of the low loading of graphene. The PANi/graphene/PANi/DWNT sheet decreased to 19.8 Ω sq^−1^ at 40 quadlayers, owing to the connectivity of the DWNT/graphene network. With the increase in the number of cycles, graphene and DWNT can help to bridge the 3D polymer-filler network, demonstrating more efficient electron transport. The quadlayer-polyelectrolyte carbon nanocomposites with 40, 50, and 60 cycles exhibited electrical conductivities of 1080 S/cm, 1025 S/cm, and 1015 S/cm, respectively, suggesting that the organic composites have a carbon content above the percolation threshold with a uniformly aligned network structure. The Seebeck coefficient of all prepared sheets is positive, showing that the sheets are all p-type. PANi/DWNT and PANi/graphene exhibited Seebeck coefficients of 95 and 25 µV/K, respectively, at 40 bilayers. The PANi/graphene/PANi/DWNT bilayer sheet exhibited a Seebeck coefficient of 130 µV/K at 40 bilayers, and its corresponding power factor was calculated to be 1825 μW/m K^−2^, which is the highest value ever reported. 

Moriarty et al. [117] prepared organic thermoelectric nanomaterials using the liquid-phase exfoliation method. Briefly, SWCNT was stabilized by poly(3,4-ethylenedioxythiophene):poly(styrene sulfonate) (PEOT:PSS) in solutions (1 mg/mL) with sonication treatment, followed by vacuum-filtering using PVDF membranes. The thickness of the membranes can be controlled by the amount of dispersion filtered. The thermal conductivity of the prepared membranes varied in a small range 0.444–0.687, with the SWCNT concentration varying from 20–95 wt %. The electrical conductivity increased with the SWCNT concentration, reaching a maximum value of 4 × 10^5^ S/m at the SWCNT concentration of 95 wt %, orders of magnitude higher than those of organic materials. The intrinsically high electrical conductivity of CNT, attributed to its highly conductive π-conjugated pathways, which can promote electron transport, is the main reason for the obtained high electrical conductivity of the nanomaterials. The Seebeck coefficient of the prepared materials changed from 26 µV/K to 14 µV/K with increased SWCNT concentrations. A small energy barrier is assumed to hinder the transport of low-energy electrons across junctions of nanotubes, resulting in an insensitive thermopower. This energy barrier for electron transport could be affected by the stabilizer of CNT. A power factor of 140 μW/m K^−2^ was obtained when the nanocomposite was incorporated by 85 wt % of SWCNT, and this value was competitive with other organic thermoelectric materials. In the end, a ZT value of 0.03 was calculated at 300 K for 40 wt % SWCNT film, demonstrating that this organic thin film can realize the energy transformation from waste heat to electricity.

**Figure 1:**
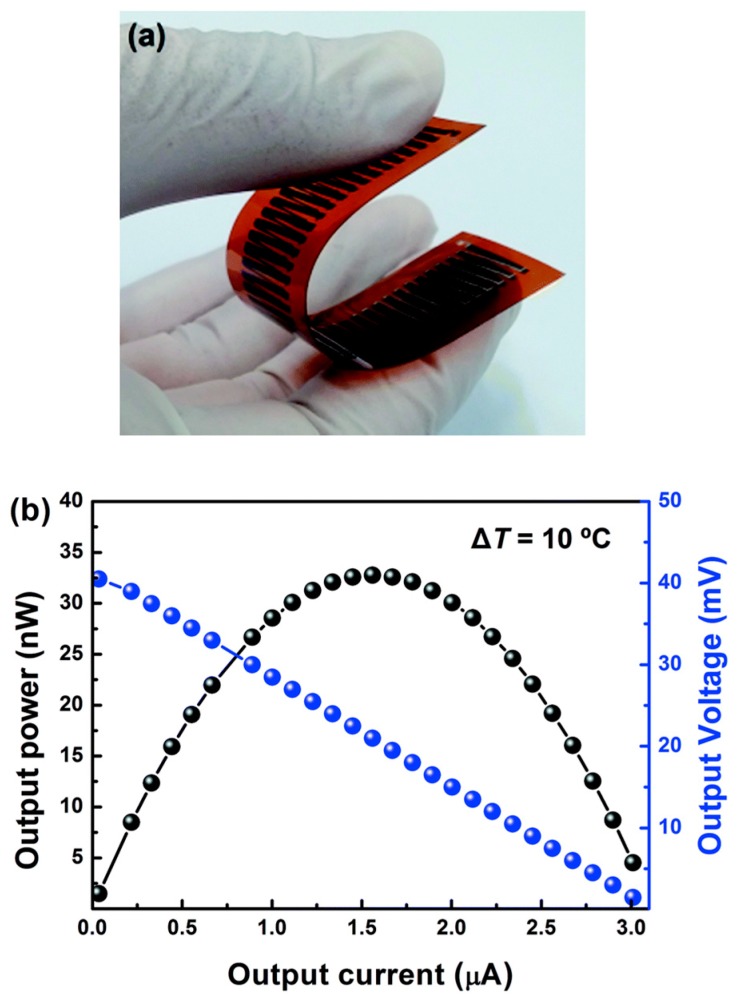
Photograph (**a**,**b**) output-power–output-current and output-voltage–output-current curves of the spray-printed flexible CNT/P3HT organic thermoelectric devices.

Hong et al. [118] prepared high-performance thermoelectric poly(3-hexylthiophene)/CNT (P3HT/CNT) nanocomposites and flexible P3HT/CNT organic thermoelectric generators by a spray-printing method. The spray-printed P3HT/CNT films exhibited excellent thermoelectric performance. Because of the p-type characteristics of both P3HT and CNT, the prepared films exhibited positive Seebeck coefficients. The Seebeck coefficient decreased with increasing CNT content, while the electrical conductivity increased. The increased power factor was attributed to the significantly increased electrical conductivity. With 50% CNT at room temperature, the electrical conductivity, Seebeck coefficient, and power factor of the nanocomposite film were determined to be 224 ± 19 S/cm, 102 ± 3 μV/K, and 231 ± 19 μW/mK^−2^, respectively. An organic thermoelectric generator (Figure 1a) composed of P3HT/CNT nanocomposite, which was spray-printed on a polyimide substrate, was prepared. Its output-power–output-current and output-voltage–output-current curves are shown in Figure 1b. The open-circuit voltage was determined to be 41.8 mV, the internal resistance was 13.5 kΩ, and the maximum output power was 32.7 nW. All these values were near the calculated values. 

Cho et al. [119] prepared a printable thermoelectric generator that was composed of organic material, graphene, PANi, and DWNT using a layer-by-layer assembly technique. The DWNT and graphene were stabilized by PEDOT:PSS, an intrinsically conductive polymer. The electrical conductivity of PANi/DWNT-PEDOT:PSS and PANi/graphene-PEDOT:PSS increased as a function of the number of deposition cycles. The PANi/graphene-PEDOT:PSS/PANi/DWNT-PEDOT:PSS quadlayers showed the highest electrical conductivity, and at 80 quadlayers, its electrical resistance decreased to 5.3 Ω/sq. The increase in the electrical conductivity with the number of deposition cycles suggests that a more interconnected network was formed for electron transport. In the case of the Seebeck coefficient, all the samples exhibited gradually increasing Seebeck coefficient with number of deposited layers. The PANi/DWNT-PEDOT:PSS, PANi/graphene-PEDOT:PSS, and PANi/graphene-PEDOT:PSS/PANi/DWNT-PEDOT:PSS quadlayers exhibited Seebeck coefficients of 58, 92, and 120 μV/K, respectively, at 80 quadlayers, which are relatively high values for organic thermoelectric materials. The power factors showed trends similar to that of the electrical conductivity. At 80 quadlayers, PANi/graphene-PEDOT:PSS showed a low power factor of 0.14 μW/mK^−2^, while PANi/DWNT-PEDOT:PSS exhibited a much higher power factor of 1230 μW/mK^−2^ at room temperature. The PANi/graphene-PEDOT:PSS/PANi/DWNT-PEDOT:PSS quadlayers exhibited the highest power factor of 2710 μW/mK^−2^, which is also the highest value reported to date. 

### 3.2. Recently Developed n-Type Organic Thermoelectric Materials

Organic thermoelectric materials in the past few decades have emerged as green energy materials. P-type thermoelectric materials have been widely reported; however, development of their n-type counterpart was relatively difficult because of the difficulties in n-type doping of organic materials. N-type thermoelectric materials are still of huge interest because of their strong demand for thermoelectric devices with light weight, flexibility, and easy processing into versatile shapes.

**Figure 2:**
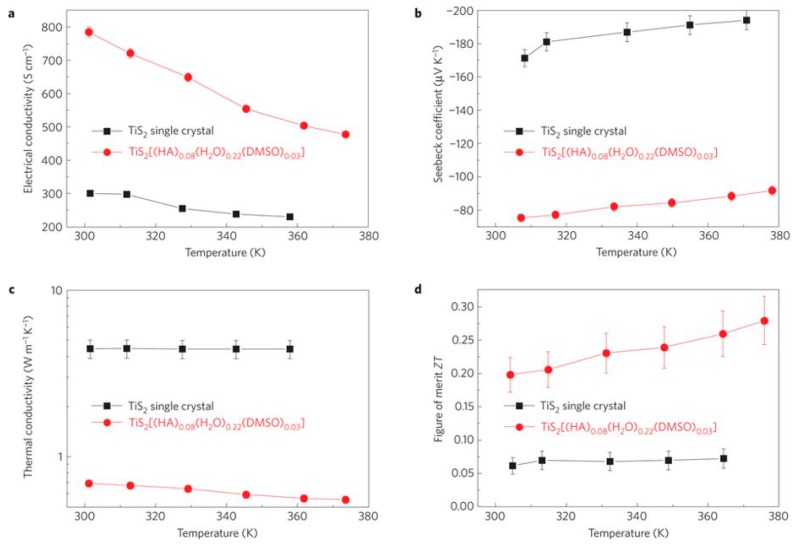
(**a**) In-plane electrical conductivity; (**b**) Seebeck coefficient; (**c**) thermal conductivity; and (**d**) in-plane *ZT*.

Wan et al. [120] prepared n-type flexible thermoelectric materials using a novel and facile electrochemical intercalation and solvent exchange approach. Briefly, TiS_2_, used as a host material, was employed as a cathode in electrochemical cells. The dissolved organic salt solution (hexylammonium (HA) chloride dissolved in dimethylsulfoxide (DMSO)) was used as the electrolyte. Given an electric potential, a hybrid-layered superlattice of organic cations and inorganic TiS_2_ was prepared. The in-plane thermoelectric results of TiS_2_[(HA)_0.08_(H_2_O)_0.22_(DMSO)_0.03_] and crystal TiS_2_ without organic intercalation were presented in Figure 2. Crystal TiS_2_ exhibited a high Seebeck coefficient of −171 µV/K despite the fact that its concentration reached as high as 3.4 × 10^20^ cm^−3^. The electrical conductivity decreased from approximately 300 S/cm to 220 S/cm with increasing the temperature from 300 to 360 K. After intercalating the hexylammonium ions, the Seebeck coefficient was significantly decreased to −78 µV/K, while the electrical conductivity increased to 790 S/cm at 300 K. This mechanism can be explained that the negative charge inside TiS_2_ was balanced by the hexylammonium ions during the electrochemical process, leading to the decreased Seebeck coefficient. The total carrier density was then increased to 7.59 × 10^20^ cm^−3^, which is almost double that in crystal TiS_2_, resulting in increased electrical conductivity. The thermal conductivity for TiS_2_[(HA)_0.08_(H_2_O)_0.22_(DMSO)_0.03_] was determined to be 0.69 W/mK, which was significantly reduced compared to that of TiS_2_ (4.45 W/mK). In the end, the calculated in-plane ZT value for TiS_2_[(HA)_0.08_(H_2_O)_0.22_(DMSO)_0.03_] reached 0.28 at 373 K, which was triple that of TiS_2_, and this value is the highest for n-type flexible thermoelectric materials.

Wu et al. [121] proposed a novel approach to fabricate an n-type SWCNT by doping diethylenetriamine (DETA) on the pristine SWCNT (p-type) and subsequent treatment by CaH_2_. The n-type DETA-CaH_2_-SWCNT was fabricated with a thickness of 13 µm. The thermoelectric performances of SWCNT, DETA-CNT, and DETA-CaH_2_-SWCNT were determined. The pristine CNT exhibited a Seebeck coefficient of 43 µV/K, while the DETA-doped CNT showed a negative Seebeck coefficient of −33.0 µV/K, and the CNT subsequently treated by CaH_2_ exhibited higher n-type characteristics with a Seebeck coefficient of −41 µV/K. The electrical conductivity of the pristine CNT which is in network form is as high as 650 S/cm, while DETA-SWCNT and DETA-CaH_2_-SWCNT show significantly decreased electrical conductivities of 230 and 165 S/cm, respectively. These values are much higher than the n-type of CNT, modified by PEI, which is usually near 39 S/cm. DETA-SWCNT and DETA-CaH_2_-SWCNT exhibited power factors of 25 and 27 μW/mK^−2^, respectively. The mechanism of the p- to n-type CNT conversion can be explained by the switching of the concentration of carrier type (electrons and holes) [122]. In pristine CNT, the holes are dominant carriers and, thus, exhibits p-type behavior. Meanwhile after doping, the Fermi level shifts upwards toward the conduction band of the SWCNTs because of the charge transfer interaction between the CNT and dopants. Thus, the Fermi level shifts much closer to the conduction band, resulting in the conversion into n-type-conducting CNT. Using the p-type and n-type thermoelectric units prepared in this study, the author assembled the modules. The pristine CNT and DETA-CaH_2_-SWCNT are employed as the p-type and n-type units, respectively. An open circuit voltage with improved temperature differences, load circuit voltage and output power with enhanced load resistance were measured. With the increase in the temperature difference, the open circuit voltages increased linearly for all thermoelectric couples. When the temperature difference values reached 55 and 110 K, the voltages for the module reached 62 and 125 mV, respectively, which are the highest performance of all organic thermoelectric modules to date. The load circuit voltage significantly increased with the load resistance, while the current decreased significantly. The output power showed a maximum when the resistance reached approximately 1500 Ω. The maximum output thermoelectric power was 649 nW when the temperature gradient was 55 K for the module containing 14 couples. 

Ferhat et al. [123] prepared a flexible inkjet-printed thermoelectric film using hybrid organic/inorganic and organic materials. The group designed a novel method to fabricate a solution-processable thermoelectric material using in-situ oxidation polymerization and interaction. Briefly, the PEDOT molecules were inserted in the nanotemplates of vanadium pentoxide (V_2_O_5_⋅nH_2_O) to increase the charge carrier concentration. The group successfully demonstrated the printability of (PEDOT)_x_V_2_O_5_ by adapting the hybrid (PEDOT)_x_V_2_O_5_ material to the inkjet printing technology. The thermoelectric performance of V_2_O_5_⋅nH_2_O and (PEDOT)_x_V_2_O_5_ were studied. The electrical conductivity increased by three orders of magnitude from 10^−4^ S/cm to 0.5 S/cm at a molar ratio of 0.6. The Seebeck coefficient decreased from −480 µV/K to −30 µV/K with the increased EPDOT molar ratio. To optimize the thermoelectric material and obtain the highest power factor, the molar ratio was tuned. The results showed that the power factor increased rapidly with added EDOT, and the highest power factor value reached 2 μW/mK^−2^ when the molar ratio was 0.1. The power factor was then decreased rapidly with increased EDOT monomer ratio. 

Cho et al. fabricated an n-type thermoelectric material by depositing polyethylenimine-stabilized double-walled CNT (DWCNT) layers and GO in a layer-by-layer fashion. PEI/GO and DWCNT-PEI/GO bilayer systems were assembled. The sequence for the bilayer deposition of PEI/GO includes the alternating adsorption of negatively charged GO and positively charged PEI. The prepared film exhibited good flexibility, able to withstand high levels of twisting, bending, and wrapping without damage or cracking. The thermoelectric properties of the PEI/GO and DWNT–PEI/GO and their thermally reduced PEI/rGO, DWNT–PEI/GO, and DWNT–PEI/rGO were studied. The electrical resistance of PEI/rGO films decreased with the increase in the number of deposition layers, while the Seebeck coefficient increased to −40 µV/K at 30 bilayers, resulting in a power factor of 0.62 μW/mK^2^. The addition of the DWCNT remarkably improved the thermoelectric performance, and the electrical conductivity reached 27.3 S/cm with a Seebeck coefficient of −30 µV/K, reaching a power factor of 2.5 μW/mK^2^ before thermal reduction. After thermal conduction, the electrical conductivity reached 460 S/cm, and the Seebeck coefficient reached −93 µV/K. A power factor of 400 μW/mK^2^ was calculated, which was the highest value for an n-type organic thermoelectric material ever reported. Because of the small thickness of the film, the thermal conductivity could not be determined. A ZT value of 0.35 was expected, assuming a thermal conductivity of 0.34 W/mK. Compared to the traditional thermoelectric materials, it is interesting to see both the electrical conductivity and Seebeck coefficient increase with the number of deposited layers. 

### 3.3. Recently Developed Organic Thermoelectric Devices

**Figure 3:**
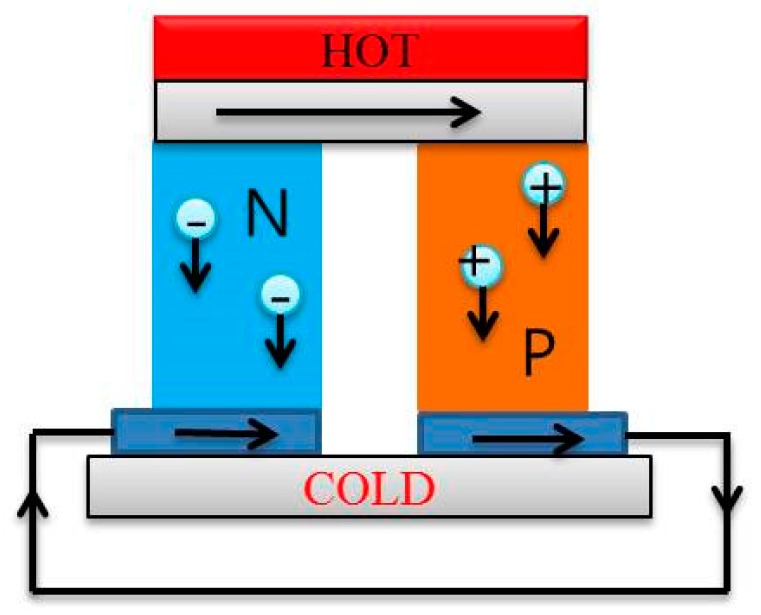
A thermoelectric module composed p-doped and n-doped semiconductors.

Thermoelectric devices or thermoelectric generators are assemblies of thermoelectric modules that are composed of n- and p-type thermoelectric materials or legs. A schematic diagram of a thermoelectric module composed of p-doped and n-doped semiconductors is shown in Figure 3. Thermoelectric generators with high thermoelectric performance have been commercially produced. In this section, some recently designed thermoelectric devices were introduced as well as their thermoelectric performance and advanced preparation technologies.

In the Hewitt study [124], an individual organic thermoelectric film composed of polyvinylidene fluoride/MWCNT that is layered into multiple modules was prepared. The power output significantly increased as the thermoelectric voltage generated by the composites was the sum of the contribution of each layer. At the same temperature, the Seebeck coefficient increased with number of layers, while the electrical conductivity decreased. Both the Seebeck coefficient and electrical conductivity for a specific sample increased with the temperature. A measurement of the power output on a 72-layer fabric was conducted as a function of load resistance at a temperature difference of 50 K. The result showed that a maximum power output of 137 nW was obtained when the load resistance was 1270 Ω. Then, the power output decreased on account of the exponentially larger load resistance. To obtain a higher power output, increasing the temperature difference and the number of conduction layers are two key factors. Theoretically, a fabric with 300 layers with a temperature difference of 100 K can reach a power output of 5 µW. Because of its lighter, cheaper, and easy-to-process material, this fabric shows promise in application to portable lightweight electronics.

Thin p- and n-type organic semiconductor films for thermoelectrical applications are fabricated by doping tetrathiotetracene (TTT) [125]. To obtain p-type materials, TTT was doped with iodine during vacuum deposition, while for n-type thin films, thermal co-deposition in vacuum of tetracyanoquinodimethane (TCNQ) and TTT was used. The Seebeck coefficient and electrical conductivity of the prepared films were characterized, and the results showed that both varied with doping level. The p-type TTT/iodide thin films exhibited a Seebeck coefficient of 63 µV/K, electrical conductivity of 130 S/s, and power factor of 0.52 μW/mK^−2^. The n-type TCNQ/TTT film exhibited a Seebeck coefficient of −75 µV/K, electrical conductivity of 57 S/s, and power factor of 0.33 μW/mK^−2^. A planar thermoelectric generator was prepared, and the electrical conductivity of the p-type material was 88 C/m, while the n-type material exhibited a conductivity of 12 S/m. The corresponding Seebeck coefficients were 62 and 118 µV/K, resulting in polymer factors of 0.33 and 0.16 μW/mK^−2^, respectively. Under ambient conditions, the thermoelectric generator exhibited a maximum power of 0.55 pW at 0.9 mV with a temperature gradient of 10 °C. After two weeks, the reduction in the power generation was less than 10%. 

Kim et al. [110] prepared CNT/PEDOT:PSS nanocomposite materials with CNT loading ranging from 10 to 50 wt %, using a wet-spinning process in a solvent/coagulation system as organic thermoelectric generators. The diameter of the prepared thermoelectric materials was 500–600 µm. The CNT/PEDOT:PSS exhibited p-type characteristics, as both CNT and PEDOT:PSS have p-type characteristics. The Seebeck coefficient of the PEDOT:PSS and its CNT-based composites was in the range of 15–18 µV/K, and the CNT concentration can significantly affect the electrical conductivity of the thermoelectrical materials. By increasing the CNT concentration, the electrical conductivity was considerably increased, because at higher CNT concentration, more CNT-CNT junctions were formed. At 50 wt % concentration of CNT, the electrical conductivity reached approximately 400 S/cm, and the power factor calculated from the electrical conductivity and Seebeck coefficient reached 10.1 ± 4.5 μW/mK^−2^ at a CNT content of 40 wt %. The n-type fibers were fabricated by post-hydrazine treatment. The results showed that the hydrazine concentration could distinctly affect the thermoelectric performance of the CNT/PEDOT:PSS materials. The Seebeck coefficient increased with the hydrazine concentration, and it was interesting to find that the Seebeck coefficient of CNT/PEDOT:PSS composites exhibited a negative value of −29 µV/K. The electrical conductivity roughly decreased with increasing hydrazine concentration. The optimized p- and n-type power factors were calculated to be 83.2 ± 6.4 μW/mK^−2^ and 35.6 ± 5.2 μW/mK^2^, respectively, at a hydrazine concentration of 0.1 wt %. An organic thermoelectric generator was assembled with twelve pairs of n- and p-type thermoelectric fibers. A temperature difference of 10 K was applied at the two ends of the thermoelectric generators, and a maximum output power of 0.430 μW was obtained. 

**Figure 4:**
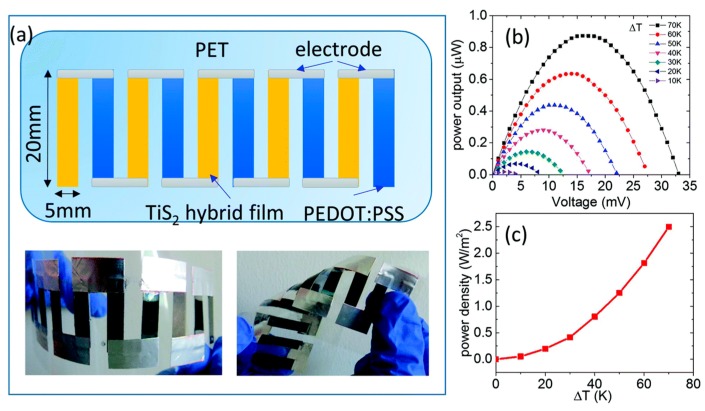
(**a**) The prototype thermoelectric devices, (**b**) the generated power output at different temperature gradients, (**c**) the calculated power density as a function of the temperature gradient.

In the study of Tian et al. [126], a facile exfoliation-and-reassembly method to fabricate a flexible n-type TiS_2_/organic hybrid film for low-temperature thermoelectric application was developed. The TiS_2_ power was ground with hexylamine in an agate mortar with a molar ratio of 1:4, resulting in a metallic brown powder. As a function of annealing time, the electrical conductivity decreased from 757 to 660 S/cm, while the Seebeck coefficient increased by 25–30%. Based on the electrical conductivity and Seebeck coefficient, the power factor was calculated as a function of annealing time. The calculated results showed that the power factor after annealing increased by 45%, and the highest power factor obtained was approximately 210 μW/mK^2^ at room temperature. The thermal conductivity of the TiS_2_ superlattice was 0.37 W/mK along the in-plane direction and 0.07 W/mK along the out-of-plane direction at 298 K. The in-plane ZT value was calculated to be 0.17 at room temperature. A prototype thermoelectric module, as shown in Figure 4, composed of five pairs of thermoelectric legs, was assembled. The resistance of this device was about 250 Ω, and the power output was shown in Figure 4b. With a temperature difference of 70 K, the devices generated a voltage of 33 mV and a power factor of 0.9 μW. At temperature gradients of 10 and 70 K, the power density was calculated to be 0.05 and 2.5 W, respectively, as shown in Figure 4c.

**Figure 5:**
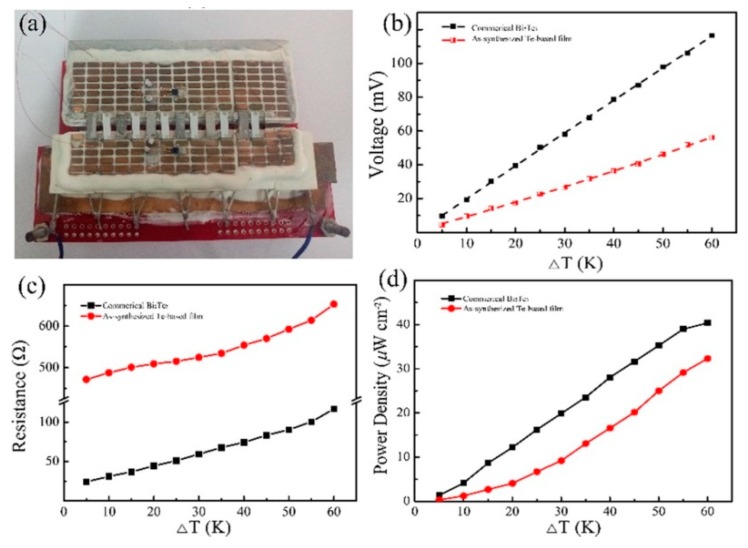
Thermoelectric device (**a**), temperature difference dependent output voltages (**b**), electrical resistances (**c**), and output powers density (**d**).

Tellurium (Te) nanomaterial has gained great attention because of its excellent thermoelectric properties; however, their practical applications have been less studied. In the Li et al. study [29], a thermoelectric device composed of n-type and p-type Te-based nanowire film legs was built for the first time. Te and Bi_2_Te_3_ nanowires were synthesized by a facial solution method. Then, a thermoelectric film containing PEDOT:PSS/Te nanowires was prepared to obtain superior thermoelectric performance and improved environmental stability. The Seebeck coefficients of the Te and Bi_2_Te_3_ films with annealing were 553 and −163 µV/K, respectively. The negative Seebeck coefficient value represents that Bi_2_Te_3_ is n-type, owing to the changes in the inherent Fermi level when the B_i_ atom is wrapped in the Te atomic surface [127]. The power factor of the prepared Bi_2_Te_3_ nanowires was found to be 78 μW m^−1^ K^−2^, exhibiting an advantage for application as thermoelectric materials. With a PEDOT:PSS content of 50 wt %, the electrical conductivity of the PEDOT:PSS/Te film reached 285 S/cm, which was almost three orders of magnitude higher than that of the Te nanowires. The Seebeck coefficient of the PEDOT:PSS/Te exhibited the opposite trend with the electrical conductivity. It is known that the Seebeck coefficient depends strongly on the density of the states of the bands close to the Fermi surface, and the PEDOT:PSS can change the Fermi level of the Te nanowires by classical interface effects and quantum size effects. A power factor of 28.5 μW m^−1^ K^−2^ was calculated for a PEDOT:PSS content of 10 wt % at room temperature for the PEDOT:PSS/Te film. A thermoelectric device composed of 6 pairs of PEDOT:PSS/Te and Bi_2_Te_3_ nanowire films was fabricated, and it is shown in Figure 5a. The output voltages of this thermoelectric device are shown in Figure 5b. It can be seen that a maximum value of 56 mV is obtained for the thermoelectric devices at a temperature difference of 60 K. The internal resistance of the fabricated device is shown in Figure 5c. The internal resistance gradually increased with the temperature difference. The calculated output power densities of this device are shown in Figure 5d. The output power densities of the device increased with the temperature difference; at a temperature difference of 60 K, a maximum output power density of 32 μW cm^−2^ was obtained.

Conductive polymers have been widely employed in developing high-performance thermoelectric materials. In the Toshima et al. study [128], a novel organic hybrid thermoelectric material was prepared for constructing a flexible thermoelectric generator using nanotechnology instead of conductive polymers with high power output. The thermoelectric material was composed of polymer complex, poly(vinyl chloride) (PVC), and CNT. The n-type semiconducting polymer complex, poly(nickel 1,1,2,2-ethenetetrathiolate) (PETT), which could help to disperse the CNT in hybrid film was successfully prepared. Besides the CNT dispersion, the nanoparticles can also enhance the carrier transport among the CNT to increase the electrical conductivity of the hybrid films, leading to an improvement in thermoelectric properties. The prepared hybrid films exhibited steady Seebeck coefficients regardless of the CNT concentration. However, the electrical conductivity was significantly affected by the CNT concentration. It was also found that the films treated with methanol exhibited higher electrical conductivity than the pristine films. A power factor of 58.6 μW m^−1^ K^−2^ was obtained. Based on a thermal conductivity value of 0.06 W/K, the maximum thermoelectric ZT value of the three-component hybrid films was anticipated to be 0.31 at 340 K. A thermoelectric device composed of 5 uni-legs was prepared by printing technology on a polyimide substrate. With an ambient temperature of 100 K, the maximum power reached 3.88 μW under matching conditions from the current–power curves in the in-plane direction. 

## 4. Conclusions and Prospects

Thermoelectric materials have drawn tremendous interest during the past few decades because of their ability to harvest energy from waste heat by green energy conversion. Although the inorganic conductors dominated the thermoelectric area up to now, their applications were limited due to their intrinsic defects. Consequently, it is urgent to develop organic polymer-based thermoelectric materials or devices that are non-toxic, convenient to process, mechanically flexible, and exhibit low thermal conductivity. In this review, parameters that could affect the thermoelectric efficiency were introduced. Some recently developed promising organic thermoelectric materials and devices were summarized regarding their thermoelectric performance, advanced preparation technologies, and influential factors. This brief review is beneficial for beginners to quickly master the fundamental knowledge of thermoelectric materials, thus inspiring them to develop more efficient thermoelectric devices. 

As mentioned in this paper, organic thermoelectric materials have exhibited various merits, but their thermoelectric efficiency is still too low to be commercially applied and produced. More work is essential to develop highly efficient thermoelectric materials for wearable green energy generators. Particularly, the adjustment of the interactions among the electric conductivity, Seebeck coefficient, and thermal conductivity should be optimized, and the fundamental understanding of the relations between structure and property should be further studied. In the quest for green energy from thermoelectric materials, more organic n-type materials should be developed. Novel designs for organic thermoelectric devices are expected. The unique energy-harvesting characteristics of organic thermoelectric material without shifting matter yield much promise as a crucial element of wearable electrical materials in future.

## Figures and Tables

**Table 1:** Chemical structures of typical organic thermoelectric polymers.

Materials	Chemical Structures
Poly(vinylidene fluoride)	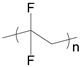
Polypyrrole	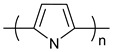
Poly(3,4-ethylenedioxythiophene)	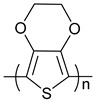
Polythiophene	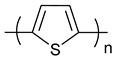
Poly(3-methylthiophene)	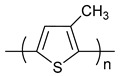
Poly(2,7-Carbazolylenevinylene)	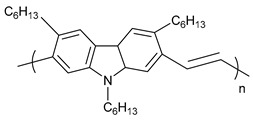
Poly(3-octylthiophene)	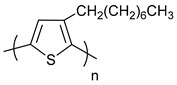
poly(3-hexylthiophene)	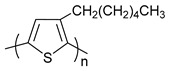
Polyaniline